# Hématome sous dural dorsal compliquant une anesthésie péridurale en chirurgie vasculaire

**DOI:** 10.11604/pamj.2014.18.231.3467

**Published:** 2014-07-20

**Authors:** Brahim Elahmadi, Almahdi Awab, Rachid El Moussaoui, Ahmed El Hijri, Abderrahim Azzouzi, Mustapha Alilou

**Affiliations:** 1 Service de Réanimation Chirurgicale, Hôpital Avicenne, CHU Ibn Sina, Rabat, Maroc

**Keywords:** Hématome sous-dural, anesthésie péridurale, chirurgie vasculaire, subdural hematoma, peridural anesthesia, vascular surgery

## Abstract

L'hématome péri médullaire est une complication rare, parfois invalidante de l'anesthésie péridurale. Il survient au décours d'une ponction difficile ou traumatique ou après une mauvaise gestion des anticoagulants. Son diagnostic est difficile parfois retardé. L'imagerie par résonance magnétique reste l'examen de choix. Le traitement est essentiellement chirurgical. Le pronostic dépend de l'importance de l'hématome et des lésions sous jacentes. Nous rapportons l'observation d'un hématome sous dural dorsal, compliquant une anesthésie péridurale en postopératoire d'un pontage aorto-bi fémoral, pour anévrysme de l'aorte abdominal sous rénale.

## Introduction

Les complications neurologiques de l'anesthésie péridurale (APD) sont rares, dont l'incidence est de 1/150000 cas en milieu chirurgical et de l'ordre de 1/500000 cas en obstétrique et ce risque s’élève à 1/1500 cas en cas d'administration d'héparine [1]. En chirurgie vasculaire et dans une revue de la littérature, nous avons relevé un seul cas d'hématome péridural en chirurgie aorto-iliaque et deux cas en chirurgie vasculaire périphérique [2–4].

## Patient et observation

**Figure 1:**
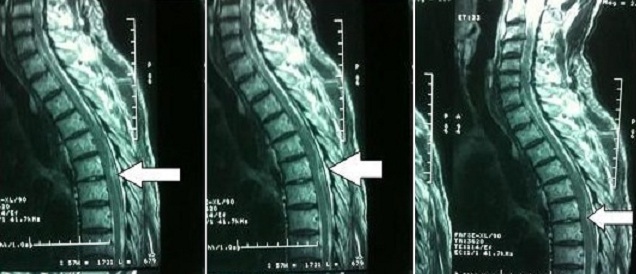
Coupes sagittales d'une IRM médullaire pondérées en T2 montrant une Collection sous durale postérieure en regard de D6-D7 sans signe de souffrance médullaire d'allure post traumatique

Nous rapportons l'observation d'un patient de 74 ans, bronchitique chronique, ayant subi un pontage aorto-bifémoral pour anévrysme de l'aorte abdominal. Le bilan biologique, notamment de la crase sanguine était normal. Pas de notion de prise d'anticoagulants en préopératoire. En vue d'une analgésie postopératoire, un cathéter péridural a été mis en place en regard de l'espace D6-D7, après deux tentatives. Le niveau sensitif a atteint D8, sans bloc moteur. L'acte chirurgical s'est déroulé sous anesthésie générale. Le clampage aortique sous rénal a duré 60 min. 30mg d'héparine sodique sont administrés au moment du clampage aortique, une heure environ après la ponction péridurale. Le saignement péropératoire a été de 1000ml et le patient est transfusé de deux culots globulaires. L'analgésie péridurale est continuée en postopératoire par la bupivacaine à 0,125%. Douze heures en postopératoire, une monoplégie inferieure droite a été constaté avec troubles sensitifs et abolition des reflex ostéotendineux. L'examen neurologique du membre controlateral était normal et il n'y avait pas de troubles sphinctériens. La bupivacaine a été arrêté et le cathéter est retiré. Un scanner du rachis dorsal était réalisé mais n’était pas concluant. Une imagerie par résonance magnétique (IRM) médullaire réalisée après 36 heures, alors que les signes cliniques commençaient à s'améliorer, a objectivé une collection sous durale postérieure en regard de D6-D7 d'allure post-traumatique, sans signes de souffrance médullaire (Figure 1). Le bilan de la crase sanguine et le taux des plaquettes étaient normaux. L'indication d'une décompression chirurgicale n'a pas été retenue devant l'amélioration du déficit moteur. L’évolution était favorable avec récupération complète du déficit sensitivomoteur en sept jours, sans séquelle neurologique.

## Discussion

Les complications neurologiques liées à l'APD sont rapportées à cinq causes principales: l'hématome compressif, l'infection, la toxicité directe des anesthésiques locaux, l'ischémie médullaire et les traumatismes directs par l'aiguille de ponction ou le cathéter péridural [5].

En chirurgie vasculaire le risque de déficit sensitivo-moteur du membre inferieur est décrit après clampage de l'aorte en particulier thoracique ou à la suite d'un clampage de l'artère iliaque supprimant une circulation de suppléance ascendante [6].

L'hématome compressif périmedullaire survient généralement au décours d'une ponction difficile ou traumatique ou après le retrait de cathéter péridural souvent associée à l'administration d'héparine [7]. Ces deux facteurs expliquent la survenu de l'hématome dans notre observation.

Le diagnostic de l'hématome périmedullaire après APD est souvent difficile, parfois retardé jusqu’à plusieurs jours. Il doit être suspecté devant un bloc sensitivo-moteur anormalement prolongé par rapport à la durée d'action de l'anesthésique local ou du morphinique utilisé, ou devant l'installation secondaire d'un déficit neurologique, douleurs dorso-lombaires ou des troubles sphinctériens. Il est confirmé par la réalisation d'une IRM ou à défaut d'une TDM médullaire.

Son traitement repose sur la réalisation en urgence, dans les six premières heures, d'une décompression médullaire chirurgicale en cas de déficit moteur lourd avec signes de compression et souffrance médullaire à l'IRM [3].

L'abstention thérapeutique peut être proposée devant un déficit neurologique léger avec un hématome peu compressif [8], sous réserve d'une surveillance clinique rapprochée et la possibilité de refaire l'IRM à tout instant. La chirurgie est alors proposée en cas d'aggravation ou de non amélioration après 48 heures.

Le pronostic neurologique dépend de l'importance de l'hématome et des lésions nerveuses sous jacentes expliquant certaines séquelles définitives même après décompression réalisée dans les meilleurs délais.

## Conclusion

Bien que l'hématome périmédullaire soit une complication rare de l'anesthésie péridurale, le pronostic neurologique mauvais fait qu'il doit être systématiquement suspecté et recherché devant la survenue de tout trouble sensitivomoteur. La prévention repose essentiellement sur le caractère non traumatique de la ponction et la bonne gestion périopératoire des anticoagulants.

## References

[1] Sophie C, Marc B (2012). Incidence et prévention des complications sévères à la péridurale. Le praticien en anesthésie réanimation..

[2] Gaudin P, Lefant D (1991). Paraplegia after epidural anesthesia for vascular surgery. Ann Fr Anesth Réanim..

[3] Osmani O, Afeiche N, Lakkis S (2000). Paraplegia after epidural anesthesia in a patient with peripheral vascular disease: case report and review of the literature with a description of an original technique for hematoma evacuation. J Spinal Disord..

[4] Martínez-Pallí G, Sala-Blanch X, Salvadó E, Acosta M, Nalda MA (1996). Epidural hematoma after epidural anesthesia in a patient with peripheral vascular disease: Case report. Reg Anesth..

[5] Renck H (1995). Neurological complications of central nerve blocks [editorial]. Acta Anaesthesiol Scund..

[6] Bromage P (1976). Paraplegia following epidural analgesia. Anaesthesia..

[7] Lena P, Teboul J, Mercier B, Bonnet B (1998). Déficit moteur des membres inférieurs et incontinence urinaire au décours d'une anesthésie péridurale. Anns Fr Anesth Réanim..

[8] Nitz P, Laubenthal H, Haller S, Mumme A, Meiser A (2008). Symptomatic epidural haematoma under therapeutic dose heparin: occurrence after removal of a peridural catheter. Anaesthesist..

